# GDNF-RET signaling and EGR1 form a positive feedback loop that promotes tamoxifen resistance via cyclin D1

**DOI:** 10.1186/s12885-023-10559-1

**Published:** 2023-02-10

**Authors:** Brooke A. Marks, Ilissa M. Pipia, Chinatsu Mukai, Sachi Horibata, Edward J. Rice, Charles G. Danko, Scott A. Coonrod

**Affiliations:** 1 Department of Biomedical and Biological Sciences, College of Veterinary Medicine, Cornell University, Ithaca, USA; 2 Baker Institute for Animal Health, College of Veterinary Medicine, Cornell University, Ithaca, USA; 3 Precision Health Program, Michigan State University, East Lansing, MI USA; 4 Department of Pharmacology and Toxicology, College of Human Medicine, Michigan State University, East Lansing, MI USA

**Keywords:** Breast cancer, Estrogen receptor alpha, Tamoxifen resistance, GDNF, RET signaling, EGR1, CCND1, Positive feedback loop

## Abstract

**Background:**

Rearranged during transfection (RET) tyrosine kinase signaling has been previously implicated in endocrine resistant breast cancer, however the mechanism by which this signaling cascade promotes resistance is currently not well described. We recently reported that glial cell-derived neurotrophic factor (GDNF)-RET signaling appears to promote a positive feedback loop with the transcription factor early growth response 1 (EGR1). Here we investigate the mechanism behind this feedback loop and test the hypothesis that GDNF-RET signaling forms a regulatory loop with EGR1 to upregulate cyclin D1 (CCND1) transcription, leading to cell cycle progression and tamoxifen resistance.

**Methods:**

To gain a better understanding of the GDNF-RET-EGR1 resistance mechanism, we studied the GDNF-EGR1 positive feedback loop and the role of GDNF and EGR1 in endocrine resistance by modulating their transcription levels using CRISPR-dCAS9 in tamoxifen sensitive (TamS) and tamoxifen resistant (TamR) MCF-7 cells. Additionally, we performed kinetic studies using recombinant GDNF (rGDNF) treatment of TamS cells. Finally, we performed cell proliferation assays using rGDNF, tamoxifen (TAM), and Palbociclib treatments in TamS cells. Statistical significance for qPCR and chromatin immunoprecipitation (ChIP)-qPCR experiments were determined using a student’s paired t-test and statistical significance for the cell viability assay was a one-way ANOVA.

**Results:**

GDNF-RET signaling formed a positive feedback loop with EGR1 and also downregulated estrogen receptor 1 (ESR1) transcription. Upregulation of GDNF and EGR1 promoted tamoxifen resistance in TamS cells and downregulation of GDNF promoted tamoxifen sensitivity in TamR cells. Additionally, we show that rGDNF treatment activated GDNF-RET signaling in TamS cells, leading to recruitment of phospho-ELK-1 to the EGR1 promoter, upregulation of EGR1 mRNA and protein, binding of EGR1 to the GDNF and CCND1 promoters, increased GDNF protein expression, and subsequent upregulation of CCND1 mRNA levels. We also show that inhibition of cyclin D1 with Palbociclib, in the presence of rGDNF, decreases cell proliferation and resensitizes cells to TAM.

**Conclusion:**

Outcomes from these studies support the hypotheses that GDNF-RET signaling forms a positive feedback loop with the transcription factor EGR1, and that GDNF-RET-EGR1 signaling promotes endocrine resistance via signaling to cyclin D1. Inhibition of components of this signaling pathway could lead to therapeutic insights into the treatment of endocrine resistant breast cancer.

**Supplementary Information:**

The online version contains supplementary material available at 10.1186/s12885-023-10559-1.

## Background

In estrogen receptor α positive (ERα+) breast cancer (BC), ERα signaling becomes overactive leading to uncontrolled replication through activation of proliferative factors, such as cyclins, and enhanced cell survival through inhibition of apoptotic factors [1]. Endocrine therapies are a common tool to inhibit specific aspects of this signaling pathway in BC patients, with tamoxifen (TAM) currently being the most common ERα+ BC therapy. However, about 20–30% of tumors from BC patients are either initially resistant to TAM therapy, also known as *de novo* resistance, or acquire TAM resistance during therapeutic treatment, resulting in tumor progression, metastasis, and increased mortality rates [2].

Several distinct processes have been implicated in TAM resistance (reviewed in [3]), with one being the upregulation of "escape pathways". One well described escape pathway is epidermal growth factor receptor (EGFR)/HER2 signaling [4–7], while a less understood pathway implicated in endocrine resistance is the RET signaling cascade [8]. RET signaling is activated by several ligands, including glial cell-derived neurotrophic factor (GDNF). Previous studies have suggested that GDNF/RET tyrosine kinase activates mitogen-activated protein kinase (MAPK) signaling components leading to endocrine resistant ERα+ breast cancer [1, 9–11] by promoting cell survival and proliferation that persists in the presence of endocrine therapies. While a relationship between GDNF-RET signaling and endocrine therapy resistance has been previously described [8, 9, 11], the mechanism remains unclear.

At the mechanistic level, GDNF is believed to activate the classical MAPK signaling pathway [12, 13], through association with the RET receptor, leading to extracellular signal-regulated kinases 1 and 2 (ERK 1/2) translocation to the nucleus and phosphorylation of downstream transcription factors (TF), such as Ets Like-1 (ELK-1). Interestingly, ELK-1 is known to bind to the early growth response 1 (EGR1) promoter, which is a known downstream target of MAPK signaling [14] and which we have previously shown to be upregulated by GDNF [9]. The TF EGR1 is activated through multiple stimuli, including growth factors, and is important for the regulation of cell growth, differentiation, and apoptosis. Additionally, EGR1 has been identified in both the suppression and progression of tumors (reviewed in [15]), with high expression of EGR1 being observed in several different cancers, such as glioma, lung, ovarian, and prostate cancer [15–22].

Moreover, EGR1 has also been linked to endocrine resistance [23], however, the importance of this TF in resistance remains controversial. Importantly, EGR1 has been proposed to be important for cell cycle progression in both MCF-7 TamS and TamR cells [24]. In further support of the role of EGR1 in BC cell proliferation, EGR1 has been shown to directly bind to the CCND1 promoter [25] and the role of the encoded cyclin D1 protein in cell cycle progression and proliferation among multiple cell types is well-described. Interestingly, ERα is also known to promote cell proliferation by directly binding to CCND1 regulatory regions [26], resulting in cell proliferation in ERα+ BC. Therefore, it is plausible that EGR1 serves as an alternative pathway for cell-cycle entry in the absence of ERα signaling due to TAM treatment.

Given the above observations, as well as previous data suggesting EGR1 upregulates GDNF [9], we hypothesized that GDNF-RET signaling promotes the upregulation of EGR1 expression, and in turn, EGR1 then upregulates GDNF expression, forming a positive feedback loop. The subsequent upregulation of CCND1 expression then promotes cell proliferation and survival in the absence of ERα signaling, leading to endocrine resistance. This current study investigates the relationship between GDNF and EGR1 and how this association sustains cell proliferation in the presence of TAM.

**Fig. 1:**
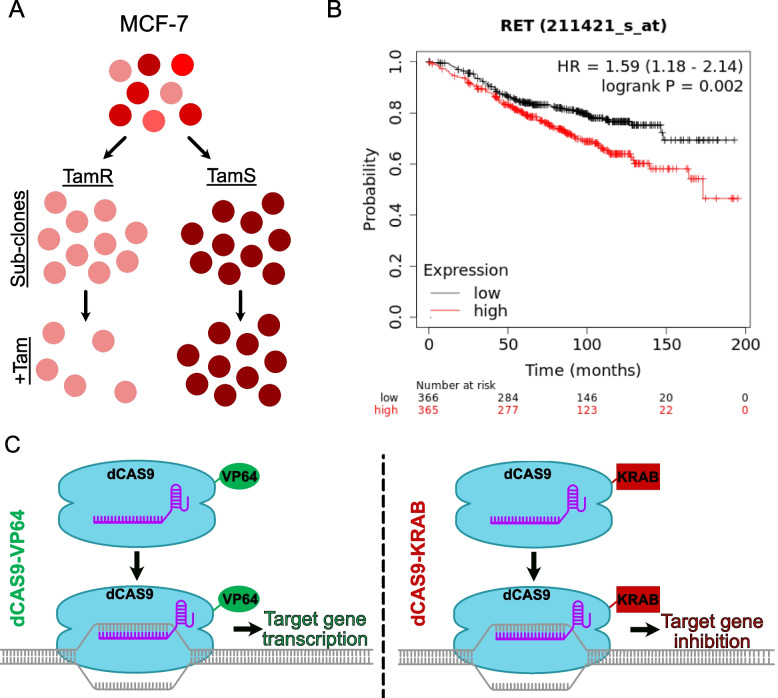
CRISPR-dCAS9 provides the ability to alter gene transcription. **A** Schematic illustration of the derivation of Tamoxifen sensitive (TamS) and resistant (TamR) MCF-7 clonal cell lines. **B** As shown by the Kaplan-Meier plotter, endocrine therapy treated ERα+ BC patients with high mRNA expression of RET (red) have decreased relapse free survival (RFS) compared to patients with low RET expression (black). **C** Components of our dCAS9 system include the modified dCAS9 protein that no longer cuts DNA, the sgRNA used by dCAS9 to detect the target sequence, and a modulating domain to either activate (VP64) or inhibit (KRAB) gene transcription at the target site

To test the hypothesis that GDNF-RET and EGR1 form a positive feedback loop to promote TAM resistance, we initially used CRISPR-dCAS9 to endogenously modulate transcription of GDNF and EGR1. We investigated how transcriptional changes of GDNF altered transcription of EGR1 and vice versa, as well as how these changes altered TAM sensitivity in MCF-7 tamoxifen sensitive (TamS) and *de novo* tamoxifen resistant (TamR) subcloned cell lines (Fig. 1A). Additionally, we activated RET signaling in TamS cells by treating cells with recombinant GDNF (rGDNF) to investigate the mechanism of resistance in a stepwise manner. To test the hypothesis that EGR1 upregulates CCND1 transcription to promote proliferation, we investigated EGR1 binding at the CCND1 promoter after rGDNF treatment. To determine the importance of cyclin D1 in the GDNF-RET-EGR1 resistance mechanism, and to investigate how inhibition of cyclin D1 alters sensitivity to TAM, we performed cell proliferation assays in TamS subclones using rGDNF, the cyclin D1-cyclin-dependent kinases 4 and 6 (CDK 4/6) complex inhibitor Palbociclib, and TAM. The consolidated results from the experiments reported here will lead to a deeper understanding of the GDNF-RET signaling mechanism and have the potential to aid in the development of novel therapies, the repurposing of current therapies, and the identification of biomarkers for the treatment of endocrine resistant breast cancer.

## Methods

### Cell lines and cell culture

TamS and TamR MCF-7 cells were a gift from Dr. Joshua LaBaer [27]. Cells were grown in Dulbecco’s Modified Eagle Medium (DMEM) with 5% fetal bovine serum (FBS) and 1X Antibiotic-Antimycotic. FBS was previously tested for tetracycline (the doxycycline derivative) activity prior to treatment in the doxycycline (DOX) inducible dCAS9 systems. Reagents used throughout this paper were: Doxycycline hyclate (2ug/ml, Sigma, cat# D9891-5G), Tamoxifen as (Z)-4-Hydroxytamoxifen (4-OHT; 1 and 5 μM, Sigma-Aldrich; Cat# H7904), Palbociclib (100 nM, Selleckchem, cat# S1579), Recombinant GDNF (10 ng/ml, Peprotech, cat# 450-10-10UG). TamS cells were transduced using a lentivirus (Mirus Trans-Lenti Transfection Reagent; Cat # MIR 6600) containing the CRISPR-dCAS9-VP64 (addgene # 50918) for the GDNF upregulated cells and CRISPR-dCAS9-TRE-VP64 (addgene #50916) for the EGR1 upregulated cells. TamR cells were transduced using the same lentivirus system, containing the CRISPR-dCAS9-KRAB (addgene # 50919) for the GDNF downregulated cells. The dCAS9-VP64 and dCAS9-KRAB plasmids contained constitutive transcription of the dCAS9-VP64/dCAS9-KRAB genes when stably inserted into the genome. The dCAS9-TRE-VP64 plasmid contained a tetracycline inducible system, where transcription of these genes were induced with DOX. The sgRNA plasmids were stably inserted into the genome using the above-mentioned lentivirus system. The sgRNA plasmid used for insertion of all sgRNAs was pLenti SpBsmBI sgRNA Hygro plasmid (addgene #62205) and sgRNA sequences are shown in Table S1. All sgRNAs were constitutively expressed. Cells were selected for successful insertion using the selection marker indicated on addgene plasmid information.

### RNA extraction and quantitative real-time PCR (qPCR)

Cells were seeded at 500,000 cells/well in a 6-well plate. RNA was collected from GDNF modulated cells 24 h later. EGR1-modulated cells were treated with +/- DOX for 24 h prior to RNA collection. For all cell lines, TRI Reagent (Molecular Research Center, Inc., cat #TR118) was used to extract RNA following the manufacturer’s instructions. cDNA was made using High-Capacity RNA-to-cDNA kit (Applied Biosystems, cat #4387406), in accordance with the manufacturer’s instructions, and then was diluted 1:5 in molecular grade water. PowerUp SYBR Green was used according to the manufacturer’s instructions (Life Technologies cat #A25741). ∆∆Ct method was used for all qPCR analyses. Data are represented as mean ±SEM (n≥3) unless otherwise mentioned. qPCR primers are found in Table S1.

### Crystal violet cell proliferation assay

Cells were seeded at 20,000 or 50,000 cells per well on 24-well or 12-well plates. GDNF-modulated cell lines were treated with 1$\mu$M TAM, 5 $\mu$M TAM, or 100% EtOH vehicle control. EGR1-modulated cell lines were treated with DOX or 70% EtOH for the vehicle control and 1$\mu$M TAM or 100% EtOH, such that each cell line had four treatment groups (+ DOX/+TAM, +DOX/-TAM, -DOX/+TAM, and -DOX/-TAM). TamS cells were treated with 10 ng/ml rGDNF, as well as 1 $\mu$M TAM, 100 nM Palbociclib, or vehicle control. Cells were grown for 5 days in their respective treatments. All experiments were performed in triplicate. After 5 days, wells were washed with phosphate-buffered saline (PBS), fixed with 500 $\mu$l of 2% PFA for 10 min, washed with PBS, stained with crystal violet (CV) stain for 10 min, and then washed three times with PBS before air drying. Once dry, 500 $\mu$l of 10% acetic acid was added to each well. The absorbance of CV in each well was detected by a TECAN plate reader (infinite M200PRO) at 595 nm. Data are represented as mean ±SEM (n≥3) unless otherwise mentioned.

### Protein analysis

Cells were seeded at 500,000 cells per plate, treated with rGDNF, and collected at each specific time point. Cells were washed with ice-cold PBS before lysing with radioimmunoprecipitation assay buffer (RIPA) buffer. Pierce protease inhibitor (Thermo Scientific, ref# A32955) and 10 mM sodium fluoride (NaF), a phosphatase inhibitor, was added fresh to the RIPA buffer. Cells were kept on ice, removed using a cell lifter, and spun down at 13,000 x g at 4℃ for 20 min. Supernatant was stored at -20℃. A micro bicinchoninic acid (BCA) assay (Thermo Scientific, cat # 23235) was used according to the manufacturer’s instructions to determine protein concentration. Protein was transferred to 0.2 $\mu$M PVDF membrane (IMMUNOBILON-P^SQ^ cat # ISEQ00010) using a Bio-Rad Trans-Blot Turbo western blot semi-dry transfer system. Membranes were cut prior to blocking to allow for EGR1, B-Actin, and GDNF protein to be probed using the same membrane. All antibodies were diluted in blocking solution and incubated overnight at 4℃. Anti-EGR1 (B-6)x (santa cruz; cat #sc-515830X) was used at 1:1000 in 5% bovine serum albumin (BSA). Anti-GDNF (AbCam, ab18956) and Anti-Beta Actin (AbCam, ab8227) antibodies were used at 1:125 and 1:5000 in 5% non-fat milk, respectively. Later detection of EGR1 and GDNF protein expression was tried using different lot numbers (Anti-GDNF Lot # GR3210959-1 and anti-EGR1 Lot # D0519) of the above antibodies, but we were unsuccessful in re-optimizing these antibodies. We are unsure if this was due to lot number changes or changes on our end. Membranes were washed in tris buffered saline with 0.1% tween20 (TBST). Secondary anti-rabbit and anti-mouse peroxidase-conjugated affiniPure antibodies from Jackson ImmunoResearch Laboratories (cat #111-035-144 and cat# 115-035-146, respectively) were diluted to 1:20000 and 1:10000, respectively, in TBST and incubated at room temperature for 1 h. Membranes were washed 3X for 20 min with TSBT and then imaged using WesternBright Quantum detection kit (cat# K-12042-D10) and Bio-Rad ChemiDoc MP.

### Chromatin immunoprecipitation

Cells were plated at 5,000,000 cells in 150 mm dishes and grown to 90–95% confluence. Cells were treated with +/- 10 ng/ml rGDNF for a specific time. Cells were then washed with PBS, cross-linked with 0.75% paraformaldehyde (Electron Microscopy Sciences, cat#15710) at room temperature for 10 min, and the crosslinking was quenched using glycine (125 mM final concentration, Fisher Scientific; cat# BP381-5). Cells were then washed twice with ice-cold PBS, harvested in 5 ml of PBS with protease and phosphatase inhibitors, spun at 1000 x g for 5 min at 4℃, and the supernatant was discarded. Pellet was resuspended in 325 $\mu$l ChIP lysis buffer (50mM HEPES-KOH pH 7.5, 140mM NaCl, 1mM EDTA pH 8, 1% Triton X-100, 0.1% Sodium Deoxycholate, 0.1% SDS, and freshly added protease inhibitors), incubated on ice for 10 min, and sonicated for 30 sec on/30 sec off until fragments were between 100 and 600 bp. 100$\mu$l of sample was diluted in 900 $\mu$l of dilution buffer **(**0.05% Tween TBS + fresh Protease inhibitor). Next, beads (see below) were added to the sample for 1 h at 4℃ to minimize non-specific background, then beads were removed and corresponding antibodies were added to each diluted aliquot and incubated overnight at 4℃ on a nutator. Anti-p-ELK-1 antibody (B4) (Santa Cruz, cat# sc-8406x) was used at 5 $\mu$g. Anti-EGR1 antibody (B6) (Santa Cruz, cat# sc-515830x) was used at 10 $\mu$g. Mouse IgG control antibody was from Diagenode (cat# C15400001). Input was kept on a nutator overnight without antibody. Immobilized Protein G (cat# 786 − 284) and Protein A (cat# 786 − 283) bead resin from G-Biosciences were used at a 70:30 (Protein G to Protein A) ratio. 40 $\mu$l of the 70:30 ratio bead resin slurry was added to samples and incubated for 2 h at 4℃ on a nutator. Beads were spun down, washed twice for 10 min at 4℃ on nutator with wash buffer (0.1% SDS, 1% Triton X-100, 2 mM EDTA pH 8, 20 mM Tris-HCL pH 8, and 150 mM NaCl) and once with high salt wash buffer (0.1% SDS, 1% Triton X-100, 2 mM EDTA pH 8, 20 mM Tris-HCL pH 8, and 500 mM NaCl). Samples were then eluted in 100 $\mu$l of fresh elution buffer (1% SDS, 100 mM NaHCO_3_, in molecular grade water) and placed at 65℃ at 1200 rpm overnight in an Eppendorf Mastercycler thermomixer. Samples were then de-crosslinked and RNA was degraded using 2 $\mu$l of 10 mg/ml Ambion RNAse (Invitrogen, cat# AM2270) for 2 h. 10 $\mu$l of 10 mg/ml Proteinase K was then added and incubated at 65℃ overnight to cleave and digest proteins in each sample. Samples were then purified using Omega Bio-Tek E.Z.N.A. Cycle Pure Kit, according to the manufacturer’s instructions, for PCR purification (cat# D6492-02) and eluted in 50 $\mu$l MQ H2O. qPCR was performed using PowerUp SYBR (applied biosystems, cat# A25742) according to the manufacturer’s instructions. 2 $\mu$l of each immunoprecipitated sample was used per reaction and the input was diluted 1:100 before use. Results were analyzed using fold enrichment.

### Kaplan-Meier plot

The Kaplan-Meier Plotter (https://kmplot.com/analysis/) mRNA gene chip for breast cancer was used. The parameters observed in the plot were as follows: response free survival, IHC and array ER positive, patients treated with endocrine therapy and without chemotherapy. Patients were split using the median to determine high and low expression levels.

### Statistics

Student’s Paired T-Test were used for qPCR and ChIP-qPCR experiments and a one-way ANOVA was used for the cell proliferation assay. Samples were normalized to controls prior to analysis. Three biological replicates were used, unless otherwise specified. In the figures, asterisks (*) denote statistical significance and data are reported as mean ± SEM. Specific p-values are indicated in the figure legends.

## Results

### High RET expression leads to a worse prognosis in endocrine therapy treated ERα+ BC patients

We first investigated the clinical importance of GDNF-RET signaling using a Kaplan-Meier plot analysis of RET expression looking at relapse-free survival (RFS) in 731 ERα+ patients who previously received endocrine therapy (Fig. 1B). We observed that high mRNA RET expression was correlated with a worse prognosis compared to patients with low RET expression. This complements previous data showing that high expression of RET ligands, including GDNF, are correlated with a lack of response in patients treated with the aromatase inhibitor letrozole [10].

### GDNF modulation alters tamoxifen sensitivity in TamS and TamR MCF-7 cell lines

To further investigate the importance of GDNF in TAM resistance, we used a CRISPR-dCAS9 construct (Fig. 1C) that targets the GDNF promoter to endogenously upregulate and downregulate GDNF in TamS and TamR cells, respectively. TamS cells express the GDNF receptor, RET tyrosine kinase, but not GDNF, and TamR cells express both RET tyrosine kinase and GDNF [10]. The dCAS9-sgRNA complex was targeted to the GDNF promoter using four constitutively transcribed sgRNA sequences. The promoter region was identified using dREG-HD [28], which shows divergent transcription, a hallmark of promoter and enhancer regions. In TamS cells, dCAS9-VP64 was stably integrated into the DNA, where it was utilized to decondense chromatin and upregulate target gene transcription (Fig. 1C). Likewise, a dCAS9-KRAB vector was stably incorporated into the TamR cell line, where it functioned to condense chromatin and inhibit target gene transcription (Fig. 1C).

**Fig. 2:**
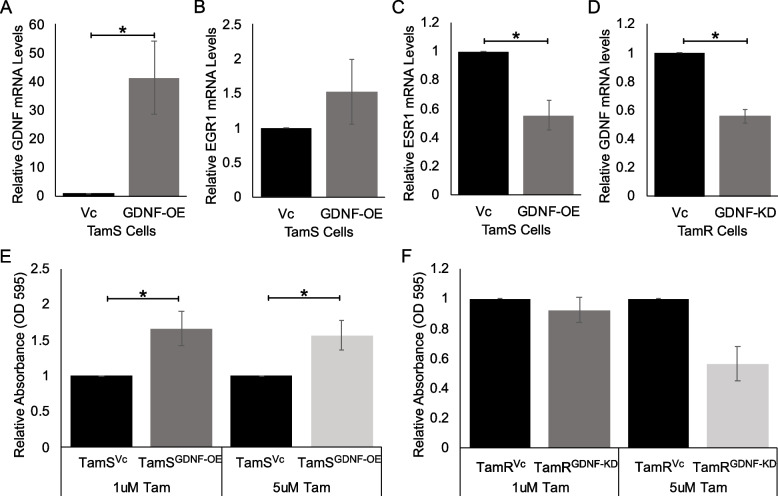
GDNF modulation using CRISPR-dCAS9 alters transcription and TAM sensitivity in TamS and TamR cells. **A** GDNF; *p *< 0.05, **B** EGR1; not significant, and (**C**) ESR1; *p *< 0.006, mRNA expression after endogenous upregulation of GDNF using CRISPR-dCAS9-VP64 system. **D** GDNF mRNA expression after endogenous downregulation of GDNF using CRISPR-dCAS9-KRAB system. **E** Cell viability of TamS^GDNF-OE^ and TamS^Vc^ cells in the presence of 1 μM and 5 μM TAM, *p *< 0.05. **F** Cell viability of TamR^GDNF-KD^ and TamR^Vc^ cells in the presence of 1 μM TAM and 5 μM (*p* < 0.065) TAM. Data in (**F**) are represented as mean ±SEM (*n*=2). Student’s paired t-test was used for statistical analyses

TamS cells containing the dCAS9-VP64 and GDNF sgRNAs (TamS^GDNF−OE^) endogenously upregulated GDNF when compared to the dCAS9-VP64 sgRNA vector control (TamS^Vc^) (Fig. 2A; *p* < 0.03). Subsequent crystal violet (CV) cell proliferation assays showed TamS^GDNF−OE^ cells proliferated and/or survived 1.5 times more than TamS^Vc^ cells when treated with 1 μM (*p* = 0.05) and 5 μM (*p* = 0.05) TAM (Fig. 2E).

Moreover, TamR cells containing the dCAS9-KRAB and GDNF sgRNAs (TamR^GDNF−KD^) endogenously downregulated GDNF transcription when compared to the dCAS9-KRAB sgRNA vector control (TamR^Vc^) (Fig. 2D, *p* < 0.001). Downregulation of GDNF in TamR^GDNF−KD^ cells promoted tamoxifen sensitivity compared to the TamR^Vc^ after 5 μM of TAM treatment (*p* < 0.065), but not after 1 μM of TAM treatment (Fig. 2G).

### Endogenous upregulation of GDNF and EGR1 alters transcription

We found that sustained upregulation of GDNF in TamS^GDNF−OE^ cells upregulated EGR1 transcription by 1.5-fold and downregulated ESR1 transcription by 0.4 fold (*p* < 0.005) (Fig. 2C and D).

**Fig. 3:**
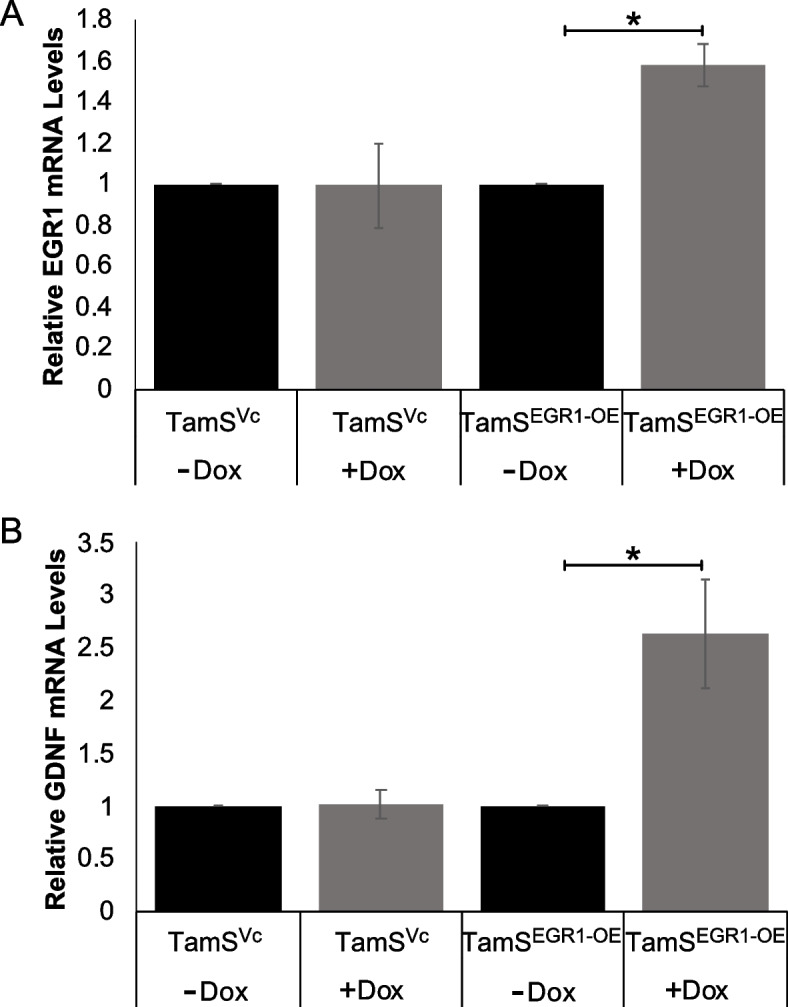
TamS^EGR1−OE^ cells upregulates GDNF transcription. **A **EGR1 (*p* < 0.005) and (**B**) GDNF (*p* < 0.05) mRNA expression levels increased after 24 h of DOX treatment in TamS^EGR1−OE^

In order to further investigate the relationship between EGR1 and GDNF signaling, we next upregulated EGR1 using six sgRNAs to target the EGR1 promoter in TamS cells (TamS^EGR1−OE^). Due to the variable nature of EGR1 expression [29], and the potential difficulty to achieve sustained upregulation, we utilized a tetracycline inducible dCAS9-VP64 system to better upregulate EGR1 expression. In this system, dCAS9 expression is dependent upon doxycycline (DOX) treatment. Treatment of TamS^EGR1−OE^ and TamS^Vc^ cells, with and without DOX for 24 h, successfully upregulated EGR1 transcription by 1.5-fold (*p* < 0.005) and GDNF by 2.5-fold (*p* < 0.05) Fig. 3A and B). Together this data suggests that GDNF and EGR1 form a positive feedback loop. Additionally, cell viability assays demonstrated that TamS^EGR1−OE^ cells proliferated 2 times faster than TamS^Vc^ cells in the presence of 1 μM TAM, however, this result was not statistically significant, potentially due to variability within replicates (Fig. S1).

### Recombinant GDNF (rGDNF) treatment activates the GDNF-EGR1 positive feedback loop in TamS cells

**Fig. 4:**
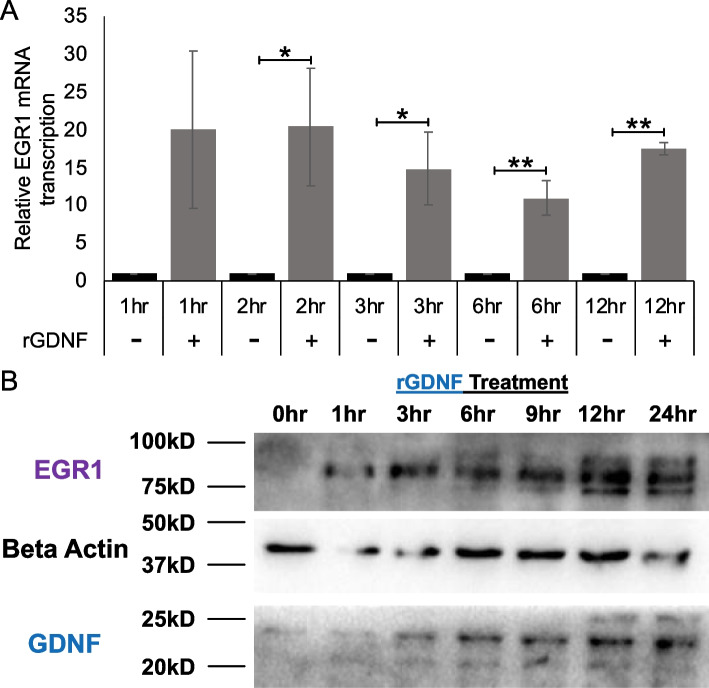
rGDNF upregulates EGR1 and GDNF. **A** EGR1 mRNA expression levels after rGDNF treatment. Student’s paired t-test was used for statistical analyses. * *p* < 0.05, ** *p* < 0.005. **B** Cropped blots of EGR1, GDNF, and B-Actin protein expression after rGDNF treatment at sequential time points. Uncropped blots are presented in Fig. S2

TamS cells express RET tyrosine kinase but do not secrete the RET ligand GDNF [10]. Addition of rGDNF activated the RET signaling pathway in vitro, thereby allowing us to test the effect of rGDNF treatment on EGR1 expression over time. Results showed that rGDNF treatment significantly upregulated EGR1 mRNA expression in TamS cells at 2, 3, 6, and 12 h post treatment (Fig. 4A). Additionally, we found that treatment of TamS cells with rGDNF also induced the expression of EGR1 protein at 1 h, with levels of EGR1 protein further increasing at subsequent timepoints. rGDNF treatment also induced endogenous GDNF expression beginning at 3 h post treatment, with protein levels appearing to increase at subsequent hours (Fig. 4B). Interestingly, higher MW protein bands were stained with the EGR1 antibody, suggesting that rGDNF treatment may also promote EGR1 phosphorylation (potentially resulting in EGR1 activation).

### GDNF upregulates EGR1 transcription through ELK1 phosphorylation

**Fig. 5:**
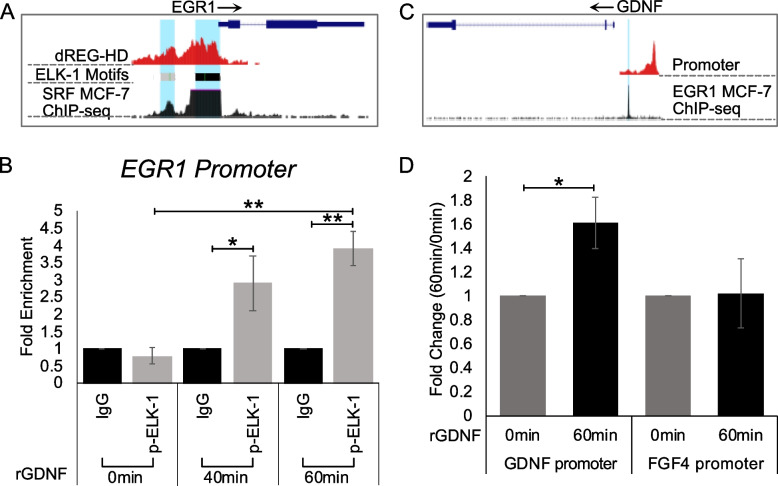
Mechanism of GDNF-EGR1 positive feedback loop. **A** UCSC genome browser data showing p-ELK-1 binding motif located at EGR1 promoter. **B** ChIP-qPCR data showing increased p-ELK-1 binding after rGDNF treatment. **C** UCSC genome browser data showing EGR1 binding motif located at the GDNF promoter. **D** ChIP-qPCR data showing increased EGR1 binding to the GDNF promoter. *p < 0.05, **p < 0.005. Student’s paired t-test was used for statistical analyses

GDNF-RET signaling has been shown to activate the MAPK signaling cascade, with ELK-1 and the transcription factor SRF (serum response factor) both being downstream targets of this kinase [9, 30]. Interestingly, through analysis of existing datasets using the UCSC genome browser, we identified an SRF ChIP-seq peak and ELK-1 binding motifs located at the EGR1 promoter in MCF-7 cells (Fig. 5A). To further understand the mechanism by which EGR1 is upregulated, we performed a Chromatin Immunoprecipitation (ChIP)-qPCR assay using an anti-phospho-ELK-1 antibody to investigate ELK-1 phosphorylation and binding at the EGR1 promoter following rGDNF treatment. Results showed that treatment of TamS cells with rGDNF resulted in a four-fold enrichment of p-ELK-1 (*p* < 0.005) at the EGR1 promoter compared to untreated cells (Fig. 5B). This result suggests that GDNF-RET signaling promotes ELK-1 binding and phosphorylation at the EGR1 promoter.

### EGR1 directly binds to the GDNF promoter

Analysis of UCSC genome browser data identified an EGR1 ChIP-seq peak at the GDNF promoter in MCF-7 cells (Fig. 5C). This data, with the above results, suggests that rGDNF treatment may promote EGR1 binding at the GDNF promoter. We tested this hypothesis and found that treatment with rGDNF resulted in a 0.6 fold enrichment of EGR1 binding to the GDNF promoter after 60 min of rGDNF treatment when compared to the 0 h control (Fig. 5D, *p* < 0.05). This enrichment was not observed in the negative control FGF4 promoter region that does not contain an EGR1 binding motif.

### The GDNF-EGR1 feedback loop likely promotes endocrine resistance by inducing CCND1 transcription

**Fig. 6:**
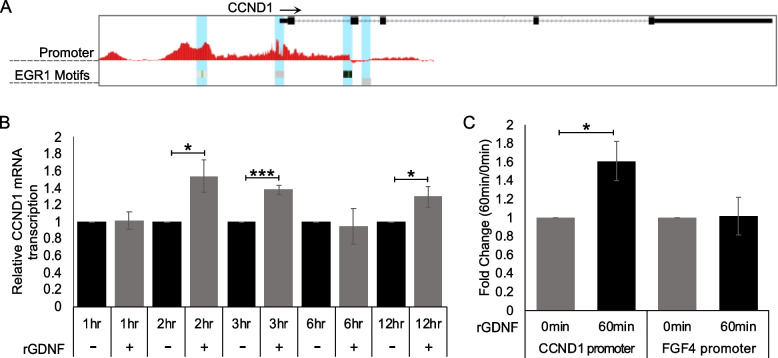
CCND1 upregulation through GDNF-RET signaling. **A** UCSC genome browser data showing EGR1 binding motif located on the CCND1 promoter. **B** CCND1 mRNA levels after 1 μM TAM treatment showing increased transcription in the presence of rGDNF after 2, 3, and 12 h. **C** ChIP-qPCR data showing increased EGR1 binding to the CCND1 promoter. Student’s paired T-Test was used for statistical analyses. * *p* < 0.05, *** *p* < 0.0005

Our study suggests that GDNF and EGR1 appear to form a positive feedback loop, however, how this feedback loop promotes TAM resistance is unknown. Analysis of UCSC genome browser data from MCF-7 cells identified four EGR1 binding motifs at the CCND1 promoter (Fig. 6A). Moreover, a previous study [25] suggests that EGR1 can bind to the CCND1 promoter to induce transcription. Therefore, we hypothesized that the GDNF-EGR1 positive feedback loop sustains EGR1 expression, and in doing so, EGR1 binds to the CCND1 promoter to induce transcription and promote cell proliferation in the presence of TAM. To test if EGR1 binds to the CCND1 promoter, we performed two experiments. First, TamS cells were treated with TAM for 24 h to inhibit cell proliferation and CCND1 transcription. Cells were then treated for various times with or without rGDNF, still in the presence of TAM. After 2, 3, and 6 h of rGDNF treatment, CCND1 transcription was significantly upregulated compared to the control (Fig. 6B, *p* < 0.05). Additionally, ChIP-qPCR was performed using an anti-EGR1 antibody after 60 min of rGDNF treatment, resulting in increased EGR1 binding to the CCND1 promoter by 1.6 fold (Fig. 6C, *p* < 0.05). This enrichment was not observed when compared to the FGF4 promoter control.

### Palbociclib inhibits rGDNF treated TamS cells and resensitizes cells to TAM

**Fig. 7:**
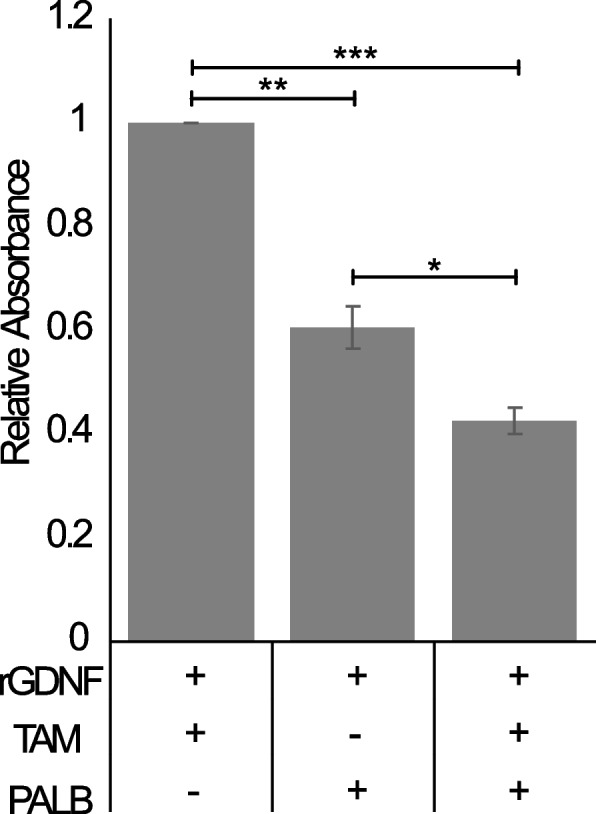
Palbociclib resensitizes TamS rGDNF treated cells to TAM. Cell viability assay shows TamS rGDNF treated cells are inhibited by Palbociclib and further inhibited by the combination of Palbociclib and TAM. One way ANOVA was used for statistical analyses. Data are represented as mean ±SEM. * *p* < 0.01; ** *p* < 0.0005; *** *p* < 0.00005

To investigate the hypothesis that (1) the GDNF-EGR1 regulatory loop promotes TAM resistance through upregulation of cyclin D1 and (2) that inhibition of this signaling mechanism promotes TAM sensitivity, we activated GDNF-RET signaling in TamS cells through rGDNF treatment and then treated TamS rGDNF cells with TAM, Palbociclib, or both. As expected in our controls, rGDNF increased growth compared to untreated cells and individual TAM and Palbociclib treatments decreased cell growth (Fig. S3). In TamS rGDNF treated cells, Palbociclib inhibited cell proliferation more than TAM ( *p* < 0.0005; Fig. 7). Additionally, when in the presence of rGDNF, TamS cells showed greater inhibition during TAM and Palbociclib combination treatment than when treated with TAM (*p* < 0.00005) or Palbociclib (*p* < 0.01) individually (Fig. 7).

## Discussion

The development of therapeutic resistance in cancer leads to progression, metastasis, and decreased overall survival. Understanding the mechanisms behind resistance are critical in developing and repurposing current therapies. In ERα+ BC, there are multiple mechanisms of resistance, some of which remain unclear and/or unknown. Among these mechanisms, RET signaling has been implicated in other cancers as well as breast cancer resistance (reviewed in [31]), however, the mechanism behind how RET signaling promotes resistance in ERα+ BC has not been previously studied. To address the clinical relevance of RET signaling, we performed a Kaplan-Meier plot analysis and found that high expression of RET was correlated with a worse prognosis for ERα+ BC patients treated with endocrine therapy, therefore further prompting this current study using TamS and TamR MCF-7 cells to elucidate the GDNF-RET signaling resistance mechanism.

Initially, we found that endogenous modulation of GDNF using the CRISPR-dCAS9 system altered tamoxifen sensitivity in ERα+ MCF-7 subclones. More specifically, TamR^GDNF−KD^ cells behaved similar to TamS cells and TamS^GDNF−OE^ cells behaved similar to TamR cells. This switch in tamoxifen sensitivity has been observed in other studies using BC cell lines, where inhibition of endocrine resistant mechanisms, like GDNF-RET-EGR1 signaling, resensitizes cells to TAM. For example, inhibition of c-Cbl, c-src, and HER2 in BT474 cells [32] and inhibition of RET and EGFR (using Gefitinib) in MCF-7 cells [8, 33], resensitized cells to TAM. This suggests that there is crosstalk between ERα signaling and mechanisms of resistance, such as GDNF-RET signaling.

Moreover, we found that in TamS^GDNF−OE^ cells, EGR1 transcription was upregulated and ESR1 transcription was downregulated, suggesting that GDNF signaling promotes endocrine resistance through simultaneously downregulating ESR1 and upregulating EGR1, thereby prompting a switch in the signaling pathway used for cell proliferation and survival. In support of this hypothesis, previous work performed in our lab has shown downregulation of ERα and phosphorylated ERα protein expression after rGDNF treatment [9]. Further investigation of this crosstalk and studies using TAM with other therapies to inhibit TAM resistance from occurring and/or resensitize tumors to TAM is needed. We also found that TamS^EGR1−OE^ cells upregulated GDNF transcription and were more resistant to TAM, though this was not significant. Taken together, the above data suggests that EGR1 upregulates GDNF transcription, and GDNF-RET signaling upregulates EGR1 transcription, supporting the idea that GDNF and EGR1 positively regulate each other.

To further investigate the GDNF-RET-EGR1 positive feedback loop mechanism, we performed kinetic studies in TamS cells. We observed that in the presence of both TAM and rGDNF treatment, EGR1 transcription was upregulated and sustained upregulation throughout treatment, suggesting that this mechanism is stable during endocrine therapy. Also, in further support of the GDNF-EGR1 positive feedback loop, EGR1 and GDNF protein expression were observed following rGDNF treatment and continued to increase throughout the rGDNF treatment period.

We next investigated how EGR1 was upregulated through GDNF-RET signaling. In the MAPK signaling cascade, ERK1/2 translocates to the nucleus where it phosphorylates multiple targets, including ELK-1. Our results show that rGDNF treatment led to increased phosphorylation of ELK-1 bound to the EGR1 promoter. These findings fit well with a previous study showing EGR1 activation through the GDNF-RET-MAPK signaling cascade [9].

As previously mentioned, upregulation of GDNF protein was observed following upregulation of EGR1 protein expression. Interestingly, UCSC genome browser data shows EGR1 MCF-7 ChIP-seq binding located at the GDNF promoter. Using ChIP-qPCR, we determined that EGR1 binds directly to the GDNF promoter following rGDNF treatment, completing the GDNF-RET-EGR1 positive feedback loop. Our findings show that in TamR MCF-7 cells, the GDNF-RET-EGR1 positive feedback loop has likely been exploited to promote TAM resistance in TamR MCF-7 cells.

We predict that this positive feedback loop represents one of multiple mechanisms used in the progression of endocrine resistant BC. This prediction is supported by the observation that RET expression is observed in 60% of patients with recurrent disease after receiving adjuvant tamoxifen therapy [8]. Further investigation into the expression of GDNF and EGR1 in RET positive BC tissue samples is needed to determine the clinical relevance of this potential resistance mechanism.

We predict that inhibition of RET signaling could represent a new therapeutic option for the treatment of endocrine resistant BC, as inhibition of RET signaling through GDNF inhibition promoted TAM sensitivity. Excitingly, the first RET inhibitor selpercatinib (Retevmo) was recently approved to treat non-small-cell lung cancer and two forms of thyroid cancer that contain RET alterations [34]. Further investigations are warranted to determine if ERα+ BC patients harbor these alterations, and if Retevmo could be a potential therapeutic, either alone or with endocrine therapies.

**Fig. 8:**
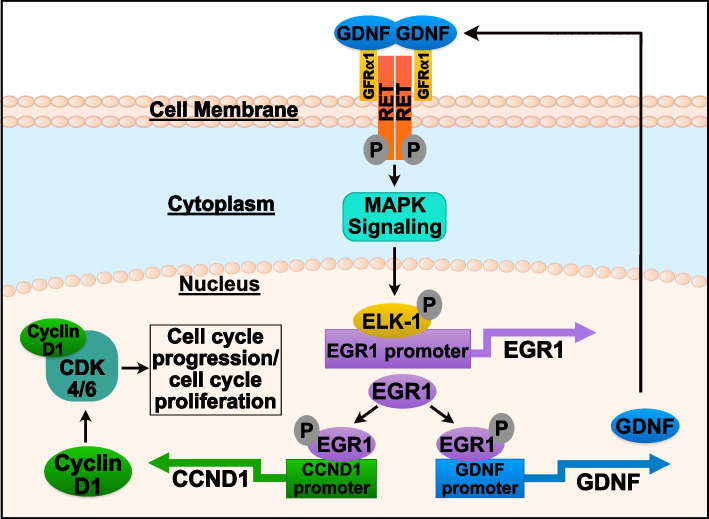
Proposed GDNF-RET-EGR1 tamoxifen resistance mechanism in ERα + breast cancer. **A** Graphical summary of the GDNF-RET-EGR1 tamoxifen resistant mechanism in ERα+ breast cancer where GDNF-RET-EGR1 signaling forms a positive feedback loop and promotes cell cycle progression through cyclin D1 upregulation

Regarding our findings with CCND1, we predicted that upon ERα inhibition GDNF-RET signaling upregulates EGR1, not only to form a positive feedback loop with GDNF, but also to promote cell proliferation in ERα+ BC patients through EGR1 binding directly to the CCND1 promoter to induce CCND1 transcription. The importance and regulation of the CCND1 protein, cyclin D1, in cell proliferation and cancer has been thoroughly investigated ([35, 36]). Our results show that in TamS cells, EGR1 not only upregulates GDNF, but also upregulates CCND1 transcription by directly binding to the CCND1 promoter after rGDNF treatment. Additionally, in TamS cells treated with TAM, CCND1 transcription was upregulated by the presence of rGDNF. This data suggests that GDNF-RET signaling promotes cell proliferation through upregulation of the transcription factor EGR1, which in turn upregulates both GDNF to promote a positive feedback loop and CCND1 to ultimately promote cell cycle progression and survival in the presence of tamoxifen (Fig. 8).

To further investigate if the GDNF-RET-EGR1 regulatory loop utilizes cyclin D1 to promote resistance, we performed cell proliferation assays following different combination treatments of rGDNF, TAM, and Palbociclib (cyclin D1-CDK4/6 inhibitor) in TamS cells. Our results showed that Palbociclib inhibited cell proliferation after rGDNF treatment and dual treatment with Palbociclib and TAM in the presence of rGDNF resulted in greater inhibition compared to both individual treatments. Our findings support that GDNF-RET signaling indirectly transcribes CCND1 and subsequently promotes cell proliferation through the cyclin D1-CDK4/6 complex and that inhibition of GDNF-RET signaling resensitizes cells to TAM.

Given our findings, one potential therapeutic option to treat GDNF-RET-EGR1 resistant BC cancer would be to inhibit both cyclin D1-CDK4/6 and ERα signaling. There are currently three CDK4/6 inhibitors available to treat advanced BC. Ibrance (Palbociclib, which was used in this study), Venzenio (Abemaciclib), and Kisqali (Ribociclib), which are all currently FDA approved for combination use with specific endocrine therapies [37–41]. Further investigation is needed to determine if this is a viable therapeutic treatment option.

## Conclusion

Overall, we observed that GDNF-RET signaling forms a positive feedback loop with EGR1, and in turn, EGR1 upregulates CCND1 to induce cell proliferation, therefore promoting tamoxifen resistance. Inhibition of this signaling pathway, through Palbociclib treatment, inhibited cell proliferation and resensitized cells to tamoxifen, resulting in further cell inhibition. This data suggests that dual treatment of cyclin D1-CDK4/6 inhibitors and endocrine therapy could have a positive impact on treating advanced endocrine resistant breast cancer patients. Further studies are needed to determine the prognostic impact of these inhibitors in GDNF-RET-EGR1 expressing breast cancer and whether these inhibitors should be used alone or in combination with current endocrine therapies.

## Supplementary Information

**Additional file 1: Table S1. **sgRNA and primer sequences. 

**Additional file 2: Figure S1.** Cell viability of TamS^EGR1-OE^ and TamS^Vc^ cells in the presence of 1uM TAM. Data are represented as mean ± SEM. **Figure S2.** Western blot from Fig. 4 showing edges of membrane. **Figure S3.** Cell viability assay in TamS cells showing the four treatment controls corresponding to Fig. 7 (vehicle, rGDNF, TAM, and Palbociclib treatment).

## Data Availability

Data files for dREG analysis have been deposited in Gene Expression Omnibus (GEO) under Accession Number GSE93229. ChIP-seq data was retrieved from publicly available MCF-7 data from the ENCODE project (doi:10.17989/ENCSR000BUX) under the GEO accession number GSM1010844.

## Abbreviations

BC: Breast cancer
ERα: estrogen receptor alpha
GDNF: Glial cell-derived neurotrophic factor
RET: rearranged during transfection
EGR1: early growth response 1
MAPK: mitogen-activated protein kinase
ERK 1/2: extracellular signal-regulated kinases 1 and 2
CRISPR: Clustered Regularly Interspaced Short Palindromic Repeats
dCAS9: Endonuclear deficient CRISPR-associated protein 9
ELK-1: Ets Like-1
TF: Transcription factor
TAM: Tamoxifen
TamS: Tamoxifen sensitive
TamR: Tamoxifen resistant
HER2: human epidermal growth factor receptor 2
EGFR: Epidermal growth factor receptor
