# Supplementary material for: GDNF-RET signaling and EGR1 form a positive feedback loop that promotes tamoxifen resistance via cyclin D1
Source: BMC Cancer. 2023 Feb 10;23:138. doi: 10.1186/s12885-023-10559-1 (PMC9912664; doi:10.1186/s12885-023-10559-1)
Supplement: Supplementary file 2 — Additional file 2: Figure S1. Cell viability of TamSEGR1-OE and TamSVc cells in the presence of 1uM TAM. Data are represented as mean ± SEM. Figure S2. Western blot from Fig. 4 showing edges of membrane. Figure S3. Cell viability assay in TamS cells showing the four treatment controls corresponding to Fig. 7 (vehicle, rGDNF, TAM, and Palbociclib treatment). [file 12885_2023_10559_MOESM2_ESM.docx]

**Figure S1.** Cell Viability of TamS^EGR1-OE^ and TamS^Vc^ cells in the presence of 1uM TAM. Data are represented as mean ±SEM.

**Figure S2.** Western blot from figure 4 showing edges of membrane.

**Figure S3.** Cell viability assay in TamS cells showing the four treatment controls corresponding to Fig. 7 (vehicle, rGDNF, TAM, and Palbociclib treatment).
